# Diagnostic, prognostic and clinical value of left ventricular radial strain to identify paradoxical septal motion in ventilated patients with the acute respiratory distress syndrome: an observational prospective multicenter study

**DOI:** 10.1186/s13054-023-04716-y

**Published:** 2023-11-02

**Authors:** Bruno Evrard, Jean-Baptiste Woillard, Annick Legras, Misylias Bouaoud, Maeva Gourraud, Antoine Humeau, Marine Goudelin, Philippe Vignon

**Affiliations:** 1 Medical-Surgical ICU, Dupuytren Teaching Hospital, 87000 Limoges, France; 2 Inserm CIC 1435, Dupuytren Teaching Hospital, 87000 Limoges, France; 3 Pharmacology & Transplantation, INSERM U1248, University of Limoges, Limoges, France; 4 Faculty of Medicine, University of Limoges, 87000 Limoges, France; 5 Department of Pharmacology, Toxicology and Pharmacovigilance, University Hospital of Limoges, Limoges, France; 6 Intensive Care Unit, Tours Teaching Hospital, Tours, France; 7 Réanimation Polyvalente, CHU Dupuytren, 87042 Limoges Cedex, France

**Keywords:** Critical care, Pulmonary heart disease, Respiratory distress syndrome, Transesophageal echocardiography

## Abstract

**Background:**

Acute cor pulmonale (ACP) is prognostic in patients with acute respiratory distress syndrome (ARDS). Identification of paradoxical septal motion (PSM) using two-dimensional echocardiography is highly subjective. We sought to describe feature-engineered metrics derived from LV radial strain changes related to PSM in ARDS patients with ACP of various severity and to illustrate potential diagnostic and prognostic yield.

**Methods:**

This prospective bicentric study included patients under protective ventilation for ARDS related to COVID-19 who were assessed using transesophageal echocardiography (TEE). Transgastric short-axis view at mid-papillary level was used to visually grade septal motion, using two-dimensional imaging, solely and combined with LV radial strain: normal (grade 0), transient end-systolic septal flattening (grade 1), prolonged end-systolic septal flattening or reversed septal curvature (grade 2). Inter-observer variability was calculated. Feature engineering was performed to calculate the time-to-peak and area under the strain curve in 6 LV segments. In the subset of patients with serial TEE examinations, a multivariate Cox model analysis accounting for new-onset of PSM as a time-dependent variable was used to identify parameters associated with ICU mortality.

**Results:**

Overall, 310 TEE examinations performed in 182 patients were analyzed (age: 67 [60–72] years; men: 66%; SAPSII: 35 [29–40]). Two-dimensional assessment identified a grade 1 and grade 2 PSM in 100 (32%) and 48 (15%) examinations, respectively. Inter-rater reliability was weak using two-dimensional imaging alone (kappa = 0.49; 95% CI 0.40–0.58; *p* < 0.001) and increased with associated LV radial strain (kappa = 0.84, 95% CI 0.79–0.90, *p* < 0.001). The time-to-peak of mid-septal and mid-lateral segments occurred significantly later in systole and increased with the grade of PSM. Similarly, the area under the strain curve of these segments increased significantly with the grade of PSM, compared with mid-anterior or mid-inferior segments. Severe acute cor pulmonale with a grade 2 PSM was significantly associated with mortality. Requalification in an upper PSM grade using LV radial strain allowed to better identify patients at risk of death (HR: 6.27 [95% CI 2.28–17.2] vs. 2.80 [95% CI 1.11–7.09]).

**Conclusions:**

In objectively depicting PSM and quantitatively assessing its severity, TEE LV radial strain appears as a valuable adjunct to conventional two-dimensional imaging.

**Supplementary Information:**

The online version contains supplementary material available at 10.1186/s13054-023-04716-y.

Acute respiratory distress syndrome (ARDS), one of the most common causes of right ventricular failure (RVF) in intensive care unit (ICU) patients, is characterized by a diffuse acute alveolar damage associated with a pulmonary vascular dysfunction and increased pulmonary vascular resistance [1]. Acute cor pulmonale (ACP) may result from this abrupt increase of RV afterload and is defined echocardiographically by the conjunction of a RV dilatation and a so-called paradoxical septal motion (PSM), which reflects the transient inversion of the interventricular pressure gradient at end-systole [2]. Several studies have shown the prognostic value of ACP in ARDS patients [3–6].

In the absence of inversed septal bulging or prolonged septal flattening, the visual diagnosis of PSM in the two-dimensional short-axis view of the heart remains challenging. Measurement of the end-systolic eccentricity index has been proposed to distinguish RV pressure from RV volume overload [7]. Nevertheless, this index is not broadly used on clinical grounds and its reproducibility is unknown. Although left ventricular (LV) radial strain provides further insights in the interventricular septum motion in patients with advanced heart failure and cardiac asynchrony or with left bundle branch block [8, 9], its ability to depict PSM has not yet been evaluated.

We tested the hypothesis that PSM could be accurately depicted by LV radial strain in ventilated ARDS patients, and help visual diagnosis in two-dimensional imaging. Accordingly, the primary objective of this study was to describe feature-engineered metrics associated with LV radial strain changes related to PSM in ARDS patients with ACP of various severity. Secondary objectives were to assess the clinical application and the prognostic value of PSM using LV radial strain when associated with conventional two-dimensional assessment.

## Study design and methods

### Population

This is an exploratory observational prospective study performed in the ICU of two University hospitals in France between March 2020 and June 2021 during the first three waves of COVID-19 in France. Eligible patients were under protective ventilation for a moderate-to-severe ARDS related to COVID-19 and underwent serial hemodynamic assessments using transesophageal echocardiography (TEE). Patients were not studied if they had a non-sinus rhythm, a chronic heart failure, or if the quality of digitally stored two-dimensional images was not adequate for accurate LV strain tracking [10]. The protocol was approved by the local Ethics Committee (#564-2022-220) which waived the need for informed consent. Non-opposition was obtained from all participants. Strobe statement checklist is provided in Additional file 1 [11].

Age, sex, Simplified Acute Physiology Score (SAPS) II, Sequential Organ Failure Assessment (SOFA) score, and comorbidities were recorded. Conventional hemodynamic and ventilatory parameters were collected at the time of TEE assessments.

### Echocardiography

TEE examinations were performed in sedated patients using a Philips EPIQ7 upper-end system equipped with a X7-1 or X8-1 transesophageal probe (Philips Healthcare, The Netherlands). In the low transesophageal four-chamber view, end-diastolic RV and LV areas were measured and their ratio computed, while interventricular septal motion was analyzed throughout the cardiac cycle in the transgastric short-axis view at the level of the mid-papillary muscle at end-expiration [12]. RV dilatation was conventionally defined by a RV/LV end-diastolic area ratio ≥ 0.6 [13]. Other TEE measurements are detailed in the Supplementary materials. All measurements were performed offline on digitally stored two-dimensional loops using the IntelliSpace CardioVascular dedicated software (Philips Medical Systems, Version 4.2.1.0, The Netherlands).

### Identification of paradoxical septal motion

Conventional visual (qualitative) identification of PSM was performed independently by two investigators with an advanced level in critical care echocardiography [14]. PSM was graded as follows: 0 in the presence of a normal septal motion; 1 if a transient end-systolic septal flattening was observed; and 2 if end-systolic septal flattening was sustained or if the septal bulging was inversed (i.e., directed toward LV cavity) at end-systole. In case of discrepancy, a third investigator with expertise in critical care echocardiography determined the presence of PSM or not, and its grade if applicable [14]. Inter-observer variability of PSM diagnosis was assessed.

**Fig. 1:**
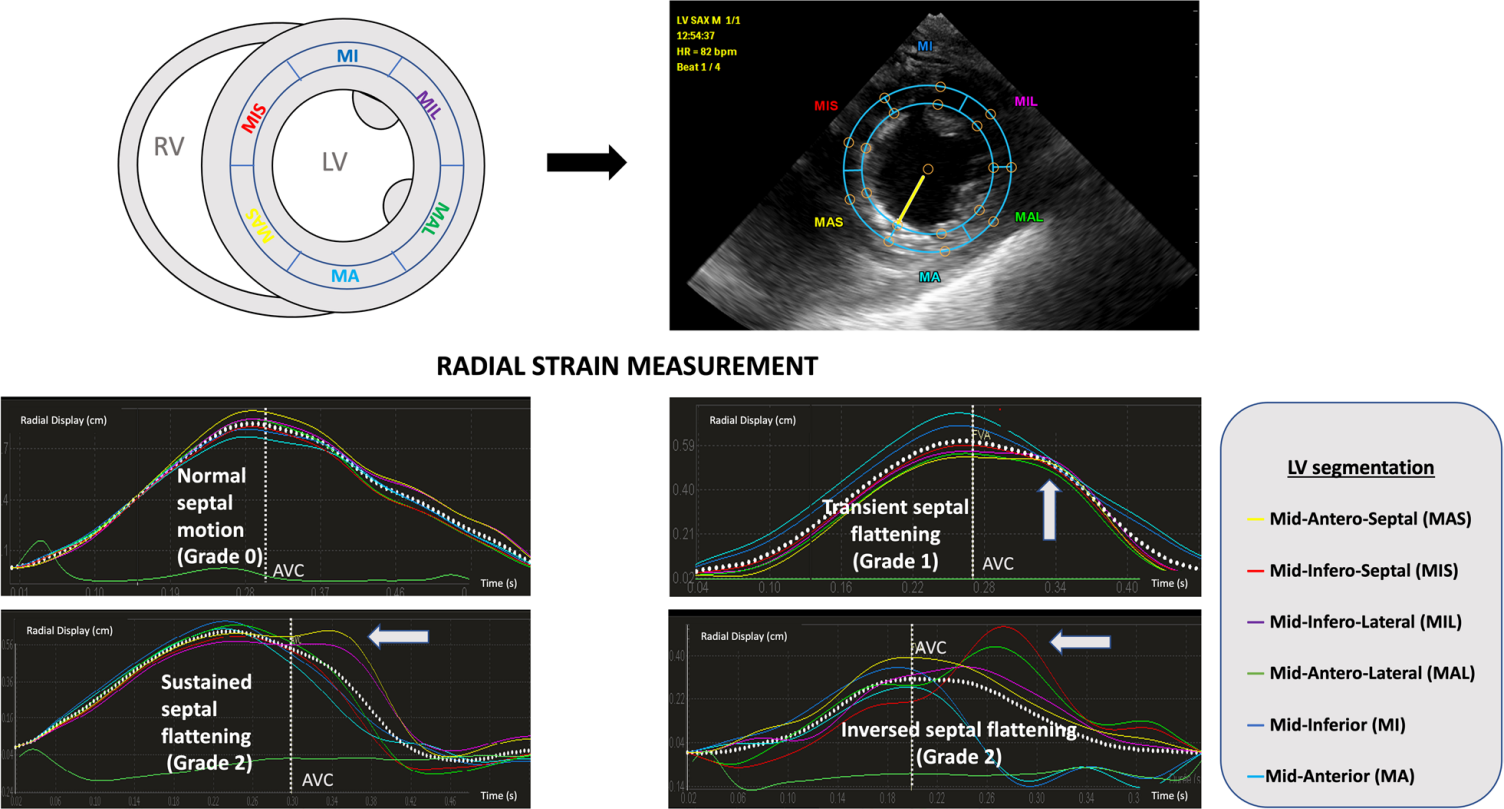
Schematic representation of left ventricular segmentation for strain analysis in the transgastric short-axis view of the heart at the mid-papillary level (upper left panel), with the overimposition of the segmental matrix on the frozen two-dimensional image (upper right panel). Each LV segment strain curve is color-encoded and the mean strain curve is displayed as a white dotted curve over-imposed on individual LV segment curves, with the time of aortic valve closure (AVC) indicated by the white vertical dotted line (lower panels). Illustrative examples of a normal septal pattern of contraction (grade 0, mid left panel) and of increasing severity of paradoxical septal motion, such as transient septal flattening (grade 1, mid right panel), sustained septal flattening (grade 2, lower left panel) or inversed septal bulging (grade 2, lower right panel) with abnormal left ventricular segmental strain curve patterns are shown (white arrows). In the presence of a normal septal motion, left ventricular segmental strain curves exhibited a uniform pattern consistent with a homogeneous regional contraction, and the peak of radial strain occurred before the aortic valve closure (mid left panel). In contrast, patients with acute cor pulmonale presented with abnormal left ventricular strain curve patterns (lower panels, white arrows). A change in the pattern of the mid-antero-septal (yellow) and/or mid-infero-septal (red) strain curves was observed: the peak was delayed (i.e., time-to-peak increased) and occurred typically after aortic valve closure. In addition, the amplitude of the delayed peak appeared related to the severity of the paradoxical septal motion when semi-quantitatively assessed using two-dimensional imaging (septal flattening vs inversed septal bulging, lower panels). Of note, the mid-infero-lateral (purple) and mid-antero-lateral (green) strain curves mirrored the abnormal pattern of left ventricular mid-antero-septal and mid-infero-septal myocardial segment, respectively (lower panels). *AVC*: aortic valve closure; *LV*: left ventricle; *RV*: right ventricle; *MIS*, mid-infero-septal segment; *MAS*, mid-antero-septal segment; *MA*; mid-anterior segment; *MAL*, mid-antero-lateral segment; *MIL*, mid-infero-lateral segment; *MI*, mid-inferior segment

The same two-dimensional digital loops were used for LV radial strain analysis. All strain measurements were performed independently by another trained operator using the QLAB 13 software (Philips, the Netherlands). The region of interest (i.e., interventricular septum) was manually determined and six LV segments were distinguished: mid-infero-septal and mid antero-septal, their opposite segments (mid-infero-lateral and mid-antero-lateral, respectively), and the remaining two segments (mid-anterior and mid-inferior) (Fig. 1). If necessary, the region of interest was adjusted to fully encompass both the mid-antero-septal and mid-infero-septal segments and their opposite segments. Aortic valve closure time was assessed visually in the three-chamber view centered on the aortic root.

Six months after this first assessment, a second evaluation of interventricular septal motion to identify potential PSM was performed by two experts on the same digital loops using both conventional two-dimensional imaging and LV radial strain. When present, PSM was graded as previously described. Loops interpretation was performed in a random order. In case of discrepancy, a third investigator with expertise in critical care echocardiography determined the presence of PSM or not, and its grade if applicable. Inter-observer variability for the diagnosis of PSM using this combined approach was also determined. This combined two-dimensional and strain imaging consensual diagnosis of PSM was used as reference.

### Data engineering

**Fig. 2:**
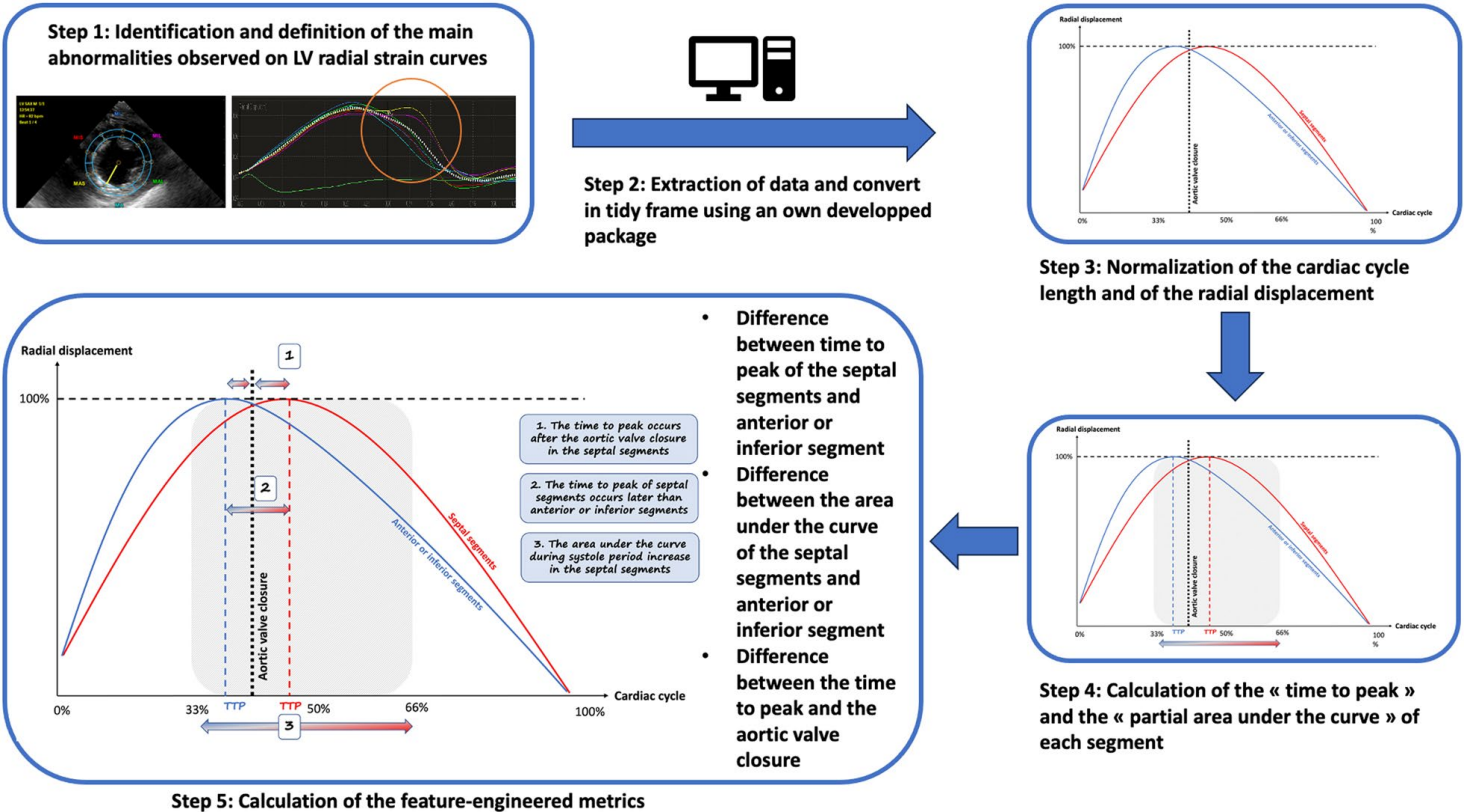
Summary of the process for data engineering. A schematic representation of the quantitative parameters calculated from left ventricular segmental strain curves is provided in the lower left panel. Both the radial displacement and time have been normalized by the maximal displacement and the duration of cardiac cycle, respectively

We first confirmed that LV radial strain curves were altered in certain segments when a PSM was present (Fig. 1) and defined the main features observed for studying (Fig. 2, lower left panel):

(i) The time to peak occurs after the aortic valve closure in the septal segments
(ii) The time to peak of septal segments occurs later than anterior or inferior segments
(iii) The area under the curve during systole period increase in the septal segments.

We then developed an R package to automatically extract the data generated with QLAB13 and converted them to a tidy data frame (https://gitlab.com/antoinehumeau/qlabraw2rectangulardata). To allow inter-individual comparisons, each measured time period was expressed as a percentage of the total length of cardiac cycle, and strain values were normalized by the maximal value measured during the cardiac cycle (Fig. 2). In each patient, the following parameters were calculated in each LV segment from strain analysis:

(i) The “time-to-peak” defined as the time lag required to reach the maximal value of strain, which was normalized by the length of cardiac cycle and expressed as a percentage.
(ii) The “partial area under segmental strain curves” which was calculated as the area under each LV segmental strain curve between 33 and 66% of the cardiac cycle length (focused time period selected on graphical examination of the strain curves where most alterations of strain pattern occurred) to standardize the measurement.

In each LV segment, these parameters were first compared between patients, according to the grade of PSM.

Because absolute value of “time-to-peak” and of the “partial area under the strain curves” could highly differ between patients, we also calculated the differences of these normalized parameters between segments in each patient. Time differences between LV segments of the time-to-peak value, of the time separating the time-to-peak and the time of aortic valve closure, and of the partial area under the strain curve were then compared between patients who were stratified according to the grade of PSM. Details for each parameter calculation are provided in the Supplementary method (Additional file 1). All steps of the above-described process of analysis is summarized in Fig. 2.

### Prognostic value assessment

To assess the prognostic value of PSM grade using LV radial strain, we conducted an exploratory analysis in a nested cohort of patients assessed with multiple TEE. Patients with only one TEE were excluded from this secondary analysis. We stratified our analysis on the severity of RV dilatation and the grade of PSM, when using two-dimensional imaging solely or in conjunction with LV radial strain. Moderate RV dilatation was defined conventionally as a RV/LV end-diastolic area ≥ 0.6 and < 1.0, and severe RV dilatation as a RV/LV end-diastolic area ≥ 1.0 [13].

### Statistical analysis

Given that no hypothesis on the expected difference in metrics can be made a priori, sample size calculation was not performed. Missing data were not replaced. Continuous data are reported as medians and interquartile ranges and were compared using Kruskall-Wallis rank-sum test and adjusted with Benjamini Hochberg method for multiple comparison. When *p*-value was ≤ 0.05, a paired comparison was performed between groups and *p*-value was adjusted with a Benjamini Hochberg test to consider the multiplicity of tests. Categorical data are reported as counts and percentages and were compared using Pearson’s Chi-square test. We determined inter-observer variability using Fleiss’ Kappa and its 95% confidence interval (CI) using bootstrapping. Inter-observer variability for the diagnosis of RV dilatation was also assessed by intra-class correlation coefficient. We assessed the diagnostic capability of PSM to predict associated RV dilatation and calculated the sensibility and the specificity of this approach. For the survival analysis in the nested longitudinal cohort, variables identified as confounding factors were selected for multivariate analysis (SAPS II, past medical history of cardiomyopathy and age). A multivariate Cox model analysis accounting for new-onset of PSM as a time-dependent variable was used to identify parameters associated with ICU mortality using conventional two-dimensional assessment alone and in association with LV radial strain [15]. A *p*-value ≤ 0.05 was considered significant. All data were generated using R software (4.2.1). R packages used are provided in the Supplementary materials (Additional file 1).

## Results

### Characteristics of population

Among the 668 patients hospitalized in the ICU for COVID-19, 195 were eligible and 13 of them could not be studied due to inadequate image quality for LV strain analysis (Additional file 1: Figure S1). Finally, 182 ventilated patients were studied (age: 67 [60–72] years; male: 66%; SAPSII: 35 [29–40]), in whom all TEE examinations (*n* = 310) were suitable for LV radial strain analysis. Only 6 patients (3%) were under vasopressors during the first TEE assessment (Table 1).

**Table 1:** General characteristics of the study population

Characteristic	*n* = 182^a^
Demographics	
Male	121 (66%)
Age (years)	67 (60, 72)
Body mass index (kg/m^2^)	30 (26, 35)
Simplified acute physiology score II	35 (29, 40)
Comorbidities	
Cardiopathy	21 (12%)
Ischemic	18 (10%)
Hypertrophic	2 (1%)
Left ventricular ejection fraction < 35%	0 (0%)
Hypertension	105 (58%)
Chronic respiratory disease	12 (6%)
Chronic renal failure	14 (8%)
Parameters on the time of the first echocardiographic assessment	
Hemodynamic parameters	
Heart rate (bpm)	82 (68, 104)
Systolic blood pressure (mmHg)	128 (110, 153)
Mean blood pressure (mmHg)	88 (76, 103)
Central venous pressure (mmHg)	9 (8, 11)
Respiratory parameters	
Tidal volume (ml/kg)	6.6 (6.0, 7.2)
Positive end-expiratory pressure (cmH2O)	12 (10, 14)
Plateau pressure (cmH2O)	25 (22, 26)
Biology	
pH	7.37 (7.30, 7.43)
PaO2/FiO2 (mmHg)	112 (87, 162)
PaCO2 (mmHg)	44 (38, 52)
Bicarbonates (mmol/L)	25 (23, 27)
Creatinin (µmol/L)	68 (56, 87)
BUN (mmol/L)	6 (5, 9)
Total bilirubin (µmol/L)	7 (5, 11)
Troponin (ng/L)	20 (14, 41)
Lactates (mmol/L)	1.4 (1.1, 1.8)
White blood Cells (G/L)	10 (7, 12)
Hemoglobin (g/dL)	12(11, 13)
Platelets (G/L)	245 (183, 303)
International normalized ratio	1.12 (1.06, 1.20)
Prothrombin time (%)	85 (76, 92)
Therapeutics	
Vasopressors	17 (9%)
Prone positioning	65 (35%)
Inhaled nitric oxyde	0 (0%)
Renal replacement therapy	1 (0.5%)
Sequential organ failure assessment	4 (3, 5)
Intensive care unit mortality	56 (31%)

^a^n (%); Median (IQR)

Interpretation of two-dimensional loops combined with LV radial strain identified a grade 1 PSM in 100 cases (32%), a grade 2 PSM in 48 cases (15%), and a normal septal motion (grade 0) in the remaining 162 examinations (52%). RV dilatation was present in 127 examinations (78%) (Table 2). This ratio significantly increased with the severity of PSM (grade 1: 0.80 [0.70–1.00] vs. grade 2: 1.10 [1.00–1.20]: *p* < 0.001) (Table 2 and Additional file 1: Figure S2). LV end-systolic eccentricity index was only slightly increased in patients with grade 1 PSM when compared to those with normal septal motion (Table 2 and Additional file 1: Figure S2), whereas it was significantly higher in patients with grade 2 PSM than in those with grade 1 (1.50 [1.30–1.75] vs. 1.10 [1.00–1.20]: *p* < 0.001). TAPSE and tricuspid S’ velocity were significantly decreased in patients with grade 2 PSM, but with median values within the normal range, while the systolic right ventriculo-atrial pressure gradient increased significantly with the grade of PSM (Table 2 and Additional file 1: Figure S2).

**Table 2:** Conventional echocardiography findings in the study population according to the systolic septal motion

Parameters	Overall (*n* = 310)	Normal septal motion (grade 0) (*n* = 162)^a^	Transient septal flattening (grade 1) (*n* = 100)^a^	Sustained septal flattening or inversed septal bulging (grade 2) (*n* = 48)^a^	*p*-value^b^	Adjusted *p*-value^c^
Left Ventricular Ejection Fraction (%)	59 (51, 67)	60 (53, 68)	58 (50, 65)	57 (51, 63)	0.2	0.2
LVOT Velocity Time Integral (cm)	21 (18, 25)	21 (18, 25)	22 (19, 26)	20 (17, 23)	0.031	0.039
RV EDA / LV EDA	0.80 (0.70, 1.00)	0.70 (0.60, 0.80)	0.80 (0.70, 1.00)	1.10 (1.00, 1.20)	< 0.001	< 0.001
End-Systolic Eccentricity Index	1.10 (1.00, 1.20)	1.00 (1.00, 1.20)	1.10 (1.00, 1.20)	1.50 (1.30, 1.75)	< 0.001	< 0.001
End-Diastolic Eccentricity Index	1.00 (1.00, 1.10)	1.00 (1.00, 1.10)	1.00 (1.00, 1.10)	1.20 (1.03, 1.40)	< 0.001	< 0.001
TAPSE (mm)	22.0 (19.0, 25.0)	22.7 (19.4, 26.0)	22.0 (20.2, 25.0)	19.0 (15.0, 24.0)	0.002	0.002
Tricuspid S’ wave (cm/s)	15.0 (12.3, 18.0)	15.0 (13.0, 18.0)	15.8 (12.6, 18.1)	12.7 (10.4, 15.1)	0.003	0.003
Vmax Tricuspid Regurgitation (cm/s)	3.00 (2.60, 3.60)	2.70 (2.35, 3.10)	3.10 (2.65, 3.70)	3.60 (3.10, 3.90)	< 0.001	< 0.001
Systolic right atrio-ventricular pressure gradient (mmHg)	36 (27, 52)	29 (22, 38)	38 (28, 55)	52 (38, 61)	< 0.001	< 0.001
RV freewall strain (%)	26 (22, 32)	27 (24, 32)	27 (23, 33)	22 (16, 28)	< 0.001	< 0.001

^a^*n* (%); Median (IQR)

^b^Fisher's exact test; Kruskal–Wallis rank sum test; Pearson's Chi-squared test

^c^False discovery rate correction for multiple testing

*RV EDA/LV EDA*: right ventricular end-diastolic area/left ventricular end-diastolic are ratio; *TAPSE*: Tricuspid annular plane systolic excursion, *LVOT*: left ventricular outflow tract

### Qualitative and quantitative assessment of LV radial strain

**Fig. 3:**
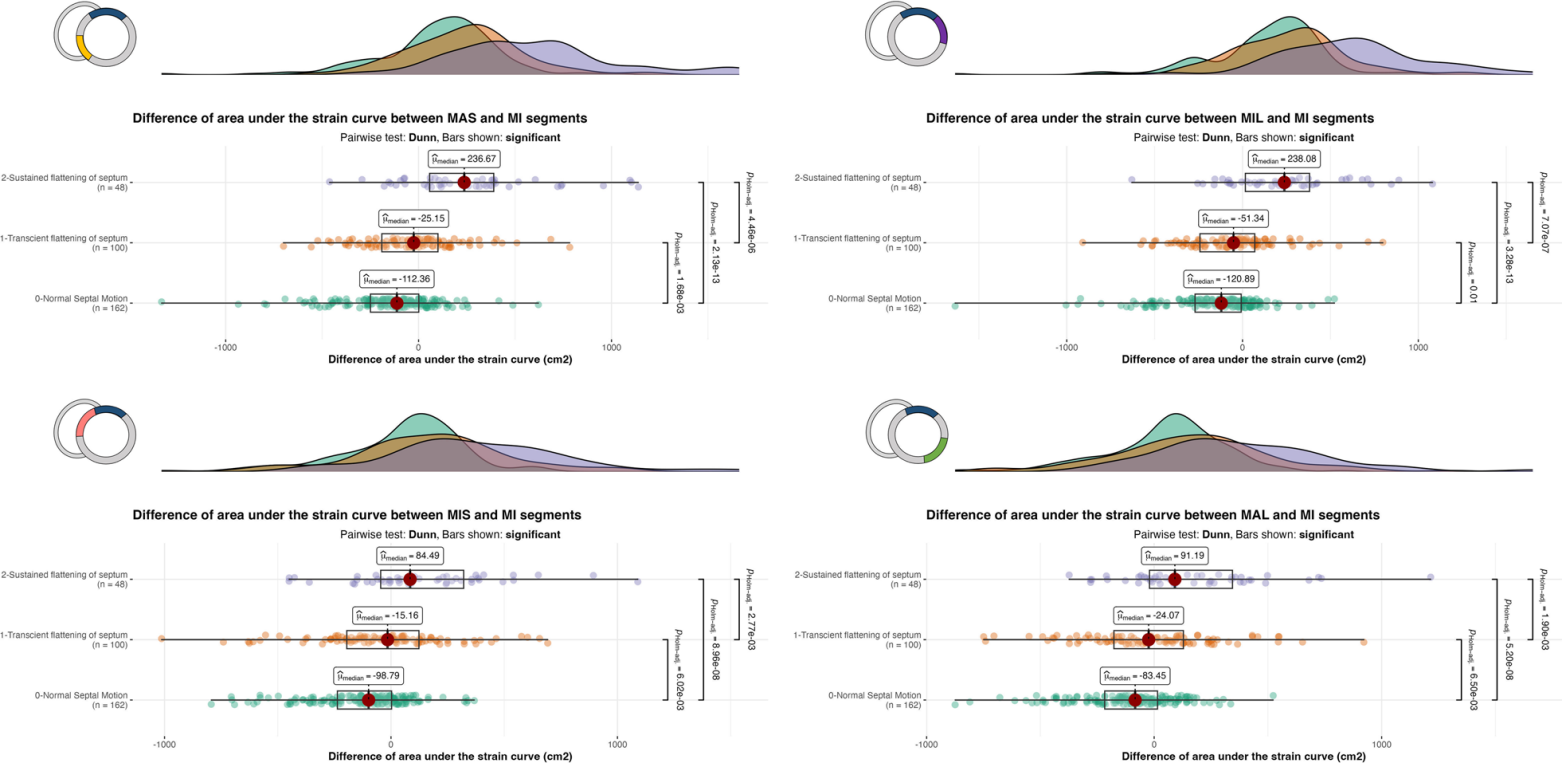
Boxplots with density plots showing the difference of area under the strain curves between the MIS and MA segments between 33 and 66% of the cardiac cycle according to the grade of septal motion (grade 0: green; grade 1: orange; grade 2: purple). In each panel, a schematic representation of LV segmentation used for the comparisons is indicated upper left. When compared to the LV MI segment, both the MAS and MIS segments exhibited increased area under the strain curve, according to the severity of paradoxical septal motion. The MIL and MAL strain curves mirrored the abnormal pattern of LV MAS and MIS segmental strain curve, respectively. P value is provided only when significant and adjusted with Benjamini–Hochberg method to take account of the multiplicity of test. *LV*: left ventricular; *MAS*: Mid-anteroseptal; *MIL*: Mid-inferolateral; *MA*: Mid-anterior; *MIS*: Mid-infero-septal; *MAL*: Mid-anterolateral; *MI*: Mid-inferior

In the presence of a PSM, LV radial strain curves exhibited abnormal patterns, as opposed to the homogeneous pattern of LV segmental strain curves observed in patients with normal septal motion (Fig. 1). When compared with LV mid-anterior or mid-inferior segments, the difference of partial area under the strain curves of mid-septal segments and their respective opposite segments (i.e., mid-antero-lateral and mid-infero-lateral segments) increased significantly with the grade of PSM (Table 3 and Fig. 3). In LV mid-septal segments and their opposite respective segments, the time-to-peak of strain occurred after aortic valve closure in patients with grade 2 PSM, when compared with their counterparts (Additional file 1: Figure S3). This was reflected by a positive difference between time-to-peak and time to aortic valve closure from the initiation of LV systole (Table 3). Accordingly, the time-to-peak of mid-septal segments and of their opposite segments increased significantly with the grade of PSM when compared with LV mid-anterior or mid-inferior segments (Table 3, Additional file 1: Figure S4). Finally, the comparison of time-to-peak and area under the partial curve in each LV segment failed to discriminate between PSM grades (Additional file 1: Table S1).

**Table 3:** Comparison of calculated parameters according to the 3 grades of paradoxical septal motion

	Normal Septal Motion Grade 0 (*n* = 162)^a^	Transient septal flattening Grade 1 (*n* = 100)^a^	Sustained septal flattening or inversed septal bulging Grade 2 (*n* = 48)^a^	*p*-value^b^	Adjusted *p*-value^c^
Difference between the partial area under the strain curve of septal or lateral segments and inferior or anterior segments (cm^2^)					
*MAS-MA*	− 110 ( − 260, 1)	− 11 ( − 155, 154)	280 (73, 492)	< 0.001	< 0.001
*MAS-MI*	− 112 ( − 250, 1)	− 25 ( − 191, 100)	237 (57, 390)	< 0.001	< 0.001
*MIS-MA*	− 84 ( − 209, 21)	− 1 ( − 174, 149)	166 ( − 26, 360)	< 0.001	< 0.001
*MIS_MI*	− 99 ( − 237, 3)	− 15 ( − 195, 123)	84 ( − 46, 321)	< 0.001	< 0.001
*MAL-MA*	− 72 ( − 208, 41)	− 7 ( − 166, 176)	140 (6, 427)	< 0.001	< 0.001
*MAL-MI*	− 83 ( − 217, 15)	− 24 ( − 177, 129)	91 ( − 20, 344)	< 0.001	< 0.001
*MIL-MA*	− 121 ( − 274, 0)	− 27 ( − 199, 109)	254 (28, 463)	< 0.001	< 0.001
*MIL-MI*	− 121 ( − 270, − 8)	− 51 ( − 242, 69)	238 (16, 381)	< 0.001	< 0.001
Difference of time between Time to peak of each segment and Aortic Valve Closure (% cycle)					
*MAS-AVC*	− 1 ( − 3, 2)	0 ( − 3, 3)	3 ( − 1, 8)	0.005	0.005
*MIS-AVC*	0 ( − 3, 4)	1 ( − 2, 6)	3 (0, 11)	0.001	0.002
*MAL-AVC*	0 ( − 3, 4)	3 ( − 1, 7)	3 (0, 10)	0.002	0.002
*MIL-AVC*	− 2 ( − 3, 2)	0 ( − 3, 3)	3 (0, 9)	< 0.001	< 0.001
*MA-AVC*	− 1 ( − 3, 4)	0 ( − 3, 2)	0 ( − 4, 2)	0.6	0.6
*MI-AVC*	− 1 ( − 3, 6)	0 ( − 3, 3)	0 ( − 4, 2)	0.5	0.5
Difference of Time to peak between septal or lateral segments and inferior or anterior segments (% cycle)					
*MAS-MA*	0 (0, 0)	0 (0, 3)	2 (0, 10)	< 0.001	< 0.001
*MAS-MI*	0 (0, 0)	0 (0, 3)	1 (0, 10)	< 0.001	< 0.001
*MIS-MA*	0 (0, 0)	0 (0, 4)	3 (0, 11)	< 0.001	< 0.001
*MIS-MI*	0 (0, 0)	0 (0, 3)	3 (0, 10)	< 0.001	< 0.001
*MAL-MA*	0 (0, 0)	0 (0, 4)	3 (0, 10)	< 0.001	< 0.001
*MAL-MI*	0 (0, 0)	0 (0, 4)	3 (0, 10)	< 0.001	< 0.001
*MIL-MA*	0 ( − 1, 0)	0 (0, 0)	3 (0, 10)	< 0.001	< 0.001
*MIL-MI*	0 ( − 2, 0)	0 (0, 0)	2 (0, 9)	< 0.001	< 0.001

^a^Median (IQR)

^b^Kruskal–Wallis rank sum test

^c^False discovery rate correction for multiple testing

*AVC*: Aortic valve closure; *MAS*: Mid-anteroseptal; *MIS*: Mid-infero-septal; *MAL*: Mid-anterolateral; *MIL*: Mid-inferolateral; *MA*: Mid-anterior; *MI*: Mid-inferior

### Diagnostic value of PSM

The inter-observer variability for the diagnosis of PSM was weak using conventional two-dimensional assessment alone (kappa = 0.49, 95% CI 0.40–0.58, *p* < 0.001), whereas it was substantially increased using associated LV radial strain visual assessment (kappa = 0.84, 95% CI 0.79–0.90, *p* < 0.001). ICC for RV/LV end-diastolic area assessment was good (ICC: 0.86, 95% CI 0.72–0.93, *p* < 0.001).

**Fig. 4:**
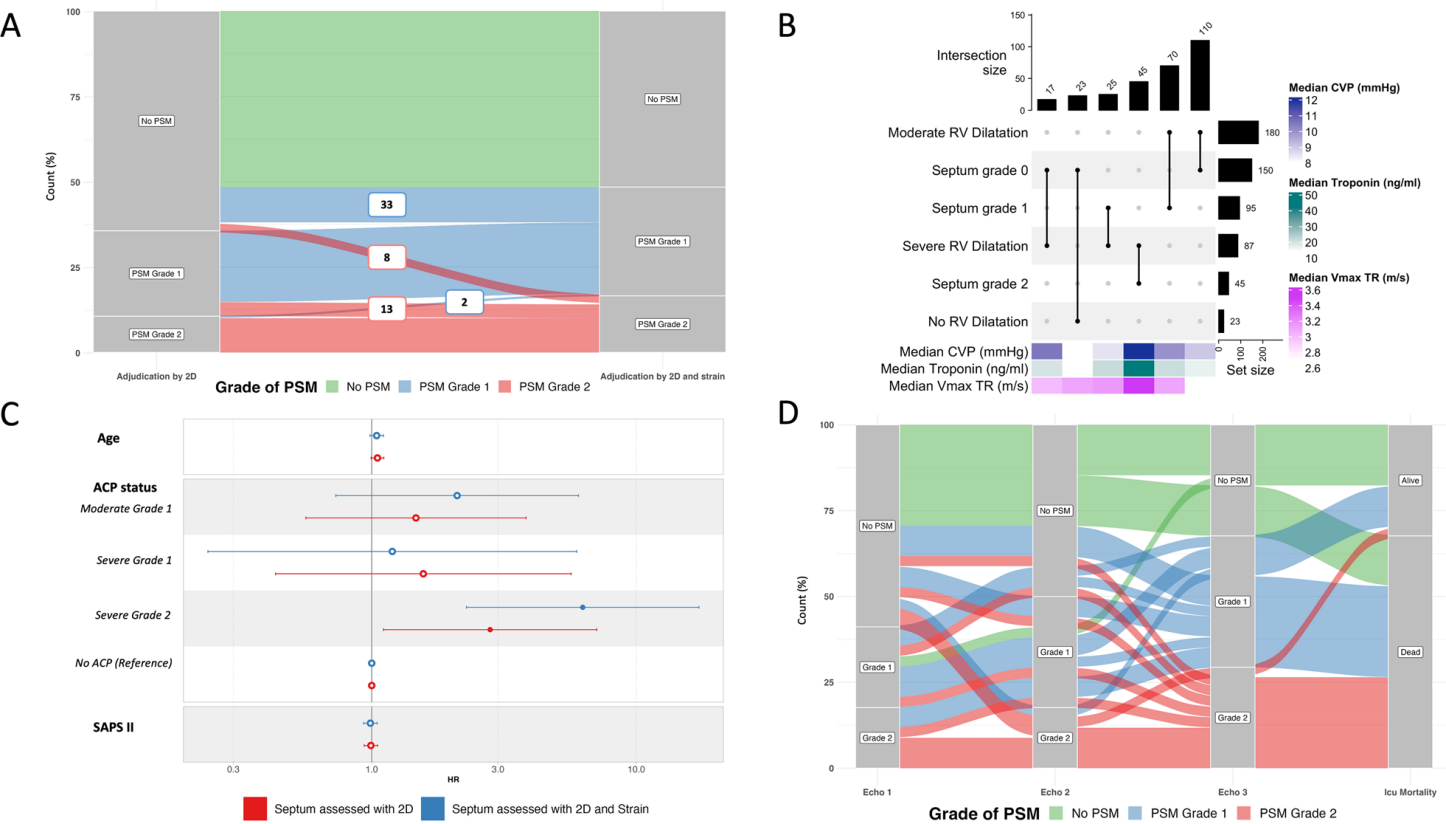
**A**. Alluvial plot depicting the variation of classification for the grade of paradoxical septal motion according to the visual diagnostic approach used (isolated conventional two-dimensional imaging vs. additional left ventricular radial strain; *n* = 310). Number in boxes correspond to the number of patients who transitioned from grade 0 or 2 to a grade 1 paradoxical septal motion (in blue) or from grade 0 or 1 to a grade 2 paradoxical septal motion (in red). **B**. Repartition of patients according to the severity of right ventricular dilatation and grade of paradoxical septal motion. Horizontal bars display the number of patients (total of 290 patients, 20 having missing values for RV dilatation assessment) with each of abnormal finding of interest, while the vertical bars depict the different combinations of RV dilatation and grade of septal motion (vertical lines connecting points). Heatmaps show median values of troponin, central venous pressure and maximal velocity of tricuspid regurgitation. **C**. Forrest plot comparing the multivariate time-dependent Cox model according to the diagnostic approach used (conventional two-dimensional assessment alone or in association with visual left ventricular radial strain interpretation). “Moderate” and “Severe” refer to the severity of right ventricular dilatation, whereas grade 1 or 2 refers to the degree of paradoxical septal motion. **D**. Alluvial plot obtained in a subset of patients (*n* = 36) who underwent a least three TEE assessments which allowed to determine the evolution of septal motion during ICU stay and outcome. *PSM*: paradoxical septal motion; *2D*: two-dimensional imaging; *TEE*: transesophageal echocardiography; *RV*: right ventricle; *CVP*: central venous pressure; *Vmax TR*: maximal velocity of tricuspid regurgitation; *ACP*: acute cor pulmonale; *SAPS II*: simplified acute physiology score 2

Thirty-three (10%) TEE studies, initially interpreted without PSM using solely conventional two-dimensional imaging, were subsequently revised as displaying a PSM grade 1, and 8 (2.5%) as PSM grade 2 when LV radial strain was used (Fig. 4A). Likewise, 13 TEE studies (4%) with an initial Grade 1 were requalified as Grade 2 PSM (Fig. 4A). Only 2 TEE studies (0.5%) were modified from Grade 2 to Grade 1 with the additional use of LV radial strain.

Grade 2 PSM was systematically associated with the presence of severe RV dilatation (specificity: 1.00, 95% CI: 0.98–1.00; sensitivity: 0.52, 95% CI: 0.41–0.63) (Fig. 4B). Likewise, grade 1 or 2 PSM was systematically associated with RV dilatation (specificity: 1.00, 95% CI: 0.85–1.00; sensitivity: 0.52, 95% CI: 0.46–0.59).

### Prognostic value of PSM

Sixty-seven patients (57%) of the initial cohort with more than one TEE assessment were included in the exploratory longitudinal analysis. Characteristics of included and excluded patients are provided in the Additional file 1: Table S2. Briefly, patients with serial TEE evaluations were more hypoxemic and had a higher mortality than their counterparts. Using the multivariate time-dependent Cox model, only severe ACP with a PSM grade 2 was significantly associated with higher risk of mortality. Using additional LV radial strain assessment, 27 patients (40%) were requalified as exhibiting a higher PSM grade. This allowed to better identify patients at high risk of death (HR: 6.27 [95% CI 2.28–17.2] vs. 2.80 [95% CI 1.11–7.09]) (Fig. 4C and Additional file 1: Table S3).

When studying the subset of patients with at least three TEE assessments, 50% of patients with PSM grade 2 on the third examination exhibited a PSM grade 0 or 1 on the previous ones, and almost all of them died (Fig. 4D). Fifty percent of patients without PSM at baseline and who developed secondarily a PSM died. Finally, among the 5 patients who died without developing PSM, none died from circulatory failure (neurological cause: *n* = 3; care withdrawal: *n*  = 2).

## Discussion

This study first describes abnormal patterns of LV radial strain in ventilated patients with ARDS and associated PSM. It shows that the magnitude of the PSM could be quantitatively assessed using parameters derived from LV segmental strain curves. It confirms the high subjectivity of visual diagnosis of PSM using conventional two-dimensional imaging in this clinical setting and suggests the additional diagnostic and prognostic value of associating LV radial strain assessment.

Accurate identification of PSM in ventilated ARDS patients is clinically relevant since ACP is associated with an increased risk of mortality [3–5] and may drive therapeutic strategies aimed at reducing RV afterload [16]. The identification of PSM relies on the visual interpretation of the two-dimensional LV short-axis view [2], which can be challenging in critically ill patients as reflected by the low inter-observer reproducibility between experts in the present study. This emphasizes the high level of subjectivity of PSM diagnosis despite optimal two-dimensional imaging quality obtained by TEE. First, the PSM may be subtle when depicted solely by a transient septal flattening at end-systole which is difficult to identify by operators who are not highly trained in critical care echocardiography [14]. Second, excessive tachycardia could make difficult the visual analysis of septal motion throughout the cardiac cycle without careful interpretation of an image loops at low speed, which is time-consuming at the bedside. End-systolic eccentricity index has been proposed as a quantitative parameter to distinguish between RV pressure and volume overload [7], but it is neither routinely used nor significantly modified in the presence of a transient septal flattening, as shown in our patients with grade 1 PSM. Moreover, the measurement of eccentricity index requires to select the specific image at end-systole which depicts the most pronounced abnormal septal motion, whereas LV radial strain automatically displays segmental wall deformation along the entire cardiac cycle. Accordingly, the diagnosis of PSM remains highly subjective, similar to the identification of LV regional wall motion abnormalities [17].

Interestingly, our results suggest that the additional use of LV radial strain may facilitate the diagnosis of PSM in clearly depicting abnormal segmental strain curve patterns, as illustrated in our patients with ACP. Facilitated PSM diagnosis allows to better identify patients at risk of developing a more severe form of ACP, which has been shown to be independently associated with ICU mortality [3, 18, 19]. In our patients with normal septal motion who were used as controls, all LV segmental strain curves exhibited a similar profile reflecting homogeneous regional contraction, and the peak of radial strain occurred before aortic valve closure. In contrast, patients with a PSM exhibited abnormal LV segmental strain curves which could be distinguished from normally contracting LV segments. Specifically, a consistent change in the morphology of either the mid-antero-septal or the mid-infero-septal strain curves was observed. The peak of the radial strain was delayed (i.e., time-to-peak from the beginning of cardiac cycle increased) and occurred at the time of, or even after aortic valve closure. This reflects a prolonged RV contraction which length exceeded that of the LV, with a resulting transient inversion of interventricular pressure gradient [20, 21]. These changes were more pronounced when the PSM was marked in two-dimensional imaging (i.e., worst abnormal strain pattern in patients with a bulging of the interventricular septum toward LV cavity at end-systole). In contrast, the peak of LV strain in the anterior or inferior segments and the strain decay occurred systematically before the aortic valve closure. Interestingly, LV mid-antero-lateral and mid-infero-lateral strain curves exhibited also an abnormal pattern which was similar to that of the mid-infero-septal and mid-antero-septal segment, respectively. This combined abnormal LV regional contraction pattern may be related to the special orientation of circumferential myocardial fibers joining the two ventricles, rather than a tethering effect [22]. In keeping with these findings, the partial area under the strain curves differed in mid-septal segments and in mid-lateral segments in patients with grade 1 and 2 PSM, when compared to the remaining LV segments (mid-anterior or mid-inferior segment).

Other echocardiography methods have been previously proposed to objectively identify PSM, such as color-encoded automatic endocardial boundary detection [23]. The main advantage of strain imaging is its accessibility and ease of use, even in the ICU setting, as shown by the small proportion of our ventilated patients (6%) who could not be enrolled in the present study due to the inability of the software to accurately track LV myocardium when imaged through the transesophageal route.

Clinical implication on mortality needs to be interpreted very cautiously because of the small sample size, which mainly focused on the most severe patients. Nevertheless, the present results confirm that severe ACP, corresponding to our patients with a grade 2 PSM, is strongly associated with mortality. This approach also takes into account the evolution during ICU stay, which provides a more relevant approach than an isolated TEE assessment on ICU admission [5, 19].

In the present study, patients with a grade 2 PSM exhibited systematically a severe RV dilatation, corresponding to a severe ACP [2]. Likewise, the presence of a grade 1 PSM was systematically associated with a RV dilatation. Alternatively, RV dilatation was associated with a PSM (irrespective of its grade) in only approximately half of patients (sensitivity ≈50%). These results need to be confirmed by further studies.

This proof-of-concept study has several limitations. The proposed metrics have been elaborated to allow quantitative comparisons of strain indices between LV segments in a given patient, and between patients for a given myocardial region, due to large overlaps of individual values between patient groups (e.g., various heart rate, variable amplitude of PSM). Nevertheless, they need to be validated in other populations at high risk of developing ACP. Rather than assessing the diagnostic capacity of cutoff values of the proposed indices derived from segmental LV strain curves, an individual machine learning predictive modeling approach would be more appropriate to best identify and quantify the presence of PSM. In addition, we purposely excluded from analysis patients with non-sinus rhythm or chronic heart failure, which are all potential confounders which presumably invalidate the proposed approach. Similarly, we did not studied patients with left bundle block branch and did not assess specifically how distal conduction abnormalities could alter segmental strain curve patterns. Nevertheless, in these patients, abnormal strain curve pattern seems to occur in early systole and apart from abnormalities related to PSM [8]. We only included patients with ARDS related to COVID-19 during the pandemic. Nevertheless, our findings can be extended to other causes of ARDS since they describe a new modality to more objectively depict the presence of a paradoxical septal motion which is the result of afterloaded RV, irrespective of its etiology. Finally, the feasibility of our approach remains to be determined when using transthoracic echocardiography, since all our ventilated ARDS patients were assessed with TEE which provided adequate imaging quality for LV radial strain analysis in more than 90% of cases.

In summary, TEE LV radial strain allows to visually help identifying abnormal patterns of contraction of mid-septal segments in ventilated ARDS patients with ACP. Parameters derived from abnormal LV segmental strain curves could provide a quantitative assessment of the magnitude of PSM. Whether a machine learning approach could allow automated, accurate identification and quantification of PSM using metrics derived from LV strain curves analysis remains to be determined.

### Supplementary Information

**Additional file 1. Supplementary study design and methods.** Echocardiography, Supplementary Data engineering and R Packages used. **Table S1.** Comparison of the different strain parameters between patients with normal septal motion and with paradoxical septal motion of different grades. **Table S2.** Comparison of characteristics between patients included and excluded from the longitudinal analysis. **Table S3.** Multivariate Time dependent Cox Model Regression using assessment only with conventional two dimensional assessment alone and with the association of LV radial strain. **Figure S1.** Flowchart of the study. **Figure S2.** Boxplots with density plots comparing right ventricles parameters stratified by the grade of septal motion. **Figure S3.** Boxplots with density plots comparing the time difference between time-to-peak and the time of aortic valve closure in each of left ventricular segment. **Figure S4.** Boxplots with density plots comparing the time difference between time-to-peak of left ventricular septal segments and anterior or inferior segments in each grade of septal motion. **Strobe statement**. **Supplementary references**.

## Data Availability

The datasets used and/or analyzed during the current study are available from the corresponding author on reasonable request.

## Abbreviations

ACP: Acute cor pulmonale
ARDS: Acute respiratory distress syndrome
COVID-19: Coronavirus disease 19
ICU: Intensive care unit
LV: Left ventricle
PSM: Paradoxical septal motion
RV: Right ventricle
SAPS II: Simplified acute physiology score II
SOFA: Sequential organ failure assessment
TEE: Transesophageal echocardiography
