# Supplementary material for: Diagnostic, prognostic and clinical value of left ventricular radial strain to identify paradoxical septal motion in ventilated patients with the acute respiratory distress syndrome: an observational prospective multicenter study
Source: Crit Care. 2023 Nov 2;27:424. doi: 10.1186/s13054-023-04716-y (PMC10623720; doi:10.1186/s13054-023-04716-y)
Supplement: Supplementary file 1 — Additional file 1. Supplementary study design and methods. Echocardiography, Supplementary Data engineering and R Packages used. Table S1. Comparison of the different strain parameters between patients with normal septal motion and with paradoxical septal motion of different grades. Table S2. Comparison of characteristics between patients included and excluded from the longitudinal analysis. Table S3. Multivariate Time dependent Cox Model Regression using assessment only with conventional two dimensional assessment alone and with the association of LV radial strain. Figure S1. Flowchart of the study. Figure S2. Boxplots with density plots comparing right ventricles parameters stratified by the grade of septal motion. Figure S3. Boxplots with density plots comparing the time difference between time-to-peak and the time of aortic valve closure in each of left ventricular segment. Figure S4. Boxplots with density plots comparing the time difference between time-to-peak of left ventricular septal segments and anterior or inferior segments in each grade of septal motion. Strobe statement. Supplementary references. [file 13054_2023_4716_MOESM1_ESM.pdf]

Diagnostic, prognostic and clinical value of left ventricular radial strain to identify paradoxical septal motion in ventilated patients with the acute respiratory distress syndrome: an observational prospective multicenter study

Bruno Evrard, MD, MSc, Jean-Baptiste Woillard, PharmD, PhD, Annick Legras , MD, Misylias Bouaoud, Maeva Gourraud, MD, Antoine Humeau, PhD, Marine Goudelin, MD, Philippe Vignon, MD, PhD

Table of contents

Supplementary study design and methods 2

Echocardiography 2

Supplementary Data engineering 2

R Packages used 4

E-Tables 5

E-Table 1: Comparison of the different strain parameters between patients with normal septal motion and with paradoxical septal motion of different grades 5

E-Table 2: Comparison of characteristics between patients included and excluded from the longitudinal analysis 6

E-Table 3: Multivariate Time dependent Cox Model Regression using assessment only with conventional two-dimensional assessment alone and with the association of LV radial strain 8

E-Figures 9

E-Figure 1: Flowchart of the study 9

E-Figure 2: Boxplots with density plots comparing right ventricles parameters stratified by the grade of septal motion. 10

E-Figure 3: Boxplots with density plots comparing the time difference between time-to-peak and the time of aortic valve closure in each of left ventricular segment 11

E-Figure 4: Boxplots with density plots comparing the time difference between time-to-peak of left ventricular septal segments and anterior or inferior segments in each grade of septal motion 12

STROBE Statement 13

Supplementary references 15

Supplementary study design and methods

Echocardiography

In the apical four-chamber view, M-mode-derived tricuspid annular plane systolic excursion (TAPSE) and maximal systolic tissue Doppler velocity recorded at the lateral aspect of the tricuspid annulus were measured. Right atrio-ventricular systolic pressure gradient was calculated using the simplified Bernouilli's equation applied to the maximal velocity of the tricuspid regurgitant jet [1]. LV outflow tract velocity-time integral (VTI) was measured in the transgastric 120° view using pulsed-wave Doppler [2].

Supplementary Data engineering

(i) The difference of area between partial area under LV segmental strain curves which was calculated as following to best capture differences which occurred between curves of the septal or lateral segments and the anterior or posterior segments:

- $pAUC\ [Mid\ anteroseptal\ segment] - pAUC[mid\ anterior\ segment]$
- $pAUC\ [Mid\ anteroseptal\ segment] - pAUC[mid\ inferior\ segment]$
- $pAUC\ [Mid\ inferoseptal\ segment] - pAUC[mid\ anterior\ segment]$
- $pAUC\ [Mid\ inferoseptal\ segment] - pAUC[mid\ inferior\ segment]$
- $pAUC\ [Mid\ anterolateral\ segment] - pAUC[mid\ anterior\ segment]$
- $pAUC\ [Mid\ anterolateral\ segment] - pAUC[mid\ inferior\ segment]$
- $pAUC\ [Mid\ inferolateral\ segment] - pAUC[mid\ anterior\ segment]$
- $pAUC\ [Mid\ inferolateral\ segment] - pAUC[mid\ inferior\ segment]$

(ii) The time difference between the time-to-peak (TTP) and the time of aortic valve closure (AVC) expressed in percentage of the cardiac cycle was calculated as follows to best capture differences which occurred between LV strain curves of the septal or lateral segments on the one hand, and of the anterior or posterior segments on the other hand:

- $TTP\ [Mid\ anteroseptal\ segment] - time\ [AVC]$
- $TTP\ [Mid\ inferoseptal\ segment] - time\ [AVC]$
- $TTP\ [Mid\ anterolateral\ segment] - time\ [AVC]$
- $TTP\ [Mid\ inferolateral\ segment] - time\ [AVC]$
- $TTP\ [Mid\ anterior\ segment] - time\ [AVC]$
- $TTP\ [Mid\ inferior\ segment] - time\ [AVC]$

(iii) The time difference of the time-to-peak (TTP) between LV segments expressed in percentage of the cardiac cycle was calculated as follows to best capture differences which occurred between LV strain curves of the septal or lateral segments on the one hand and of the anterior or posterior segments on the other hand:

- $TTP\ [Mid\ anteroseptal\ segment] - TTP[mid\ anterior\ segment]$
- $TTP\ [Mid\ anteroseptal\ segment] - TTP[mid\ inferior\ segment]$
- $TTP\ [Mid\ inferoseptal\ segment] - TTP[mid\ anterior\ segment]$
- $TTP\ [Mid\ inferoseptal\ segment] - TTP[mid\ inferior\ segment]$
- $TTP\ [Mid\ anterolateral\ segment] - TTP[mid\ anterior\ segment]$
- $TTP\ [Mid\ anterolateral\ segment] - TTP[mid\ inferior\ segment]$
- $TTP\ [Mid\ inferolateral\ segment] - TTP[mid\ anterior\ segment]$

- $TTP\ [Mid\ inferolateral\ segment] - TTP[mid\ inferior\ segment]$

R Packages used

Processing of data:

- "tidyverse"
- "qlabRaw2RectangularData" [3]

Statistical analysis:

- Time dependent Cox model: "survival"
- Inter rater reliability: "irr" and "boost"
- Area under the curves: "pROC"
- Other: "epiR"
- Statistic summary: "gtsummary" [4]

Figures:

- "GGally"
- "ComplexHeatmap" [5]
- "Ggalluvial" [6]
- "Ggstatsplot" [7]

E-Tables

E-Table 1: Comparison of the different strain parameters between patients with normal septal motion and with paradoxical septal motion of different grades

	Normal Septal Motion, N = 162[1]	Transient septal flattening N = 100[1]	Sustained septal flattening or inversed septal bulging, N = 48[1]	p-value[2]	Adjusted p-value[3]
**Time for Aortic valve closure (% cycle)**	39 (36, 43)	39 (35, 42)	39 (36, 43)	0.3	0.4
**Time to peak for each segment (% cycle)**					
MAS	39 (34, 45)	39 (35, 43)	42 (38, 48)	0.041	0.2
MIS	39 (35, 45)	39 (36, 45)	41 (37, 52)	0.2	0.3
MAL	39 (35, 46)	40 (36, 46)	44 (37, 47)	0.3	0.3
MIL	38 (34, 44)	38 (35, 43)	42 (36, 47)	0.049	0.2
MA	39 (35, 46)	37 (35, 42)	38 (31, 44)	0.12	0.2
MI	39 (35, 47)	38 (35, 42)	38 (32, 44)	0.13	0.2
**Area under the strain curve of each segment between 33 and 66% of cycle (cm^2)**					
MAS	2,567 (2,223, 2,801)	2,556 (2,230, 2,788)	2,738 (2,371, 2,946)	0.12	0.2
MIS	2,535 (2,275, 2,833)	2,535 (2,222, 2,767)	2,599 (2,203, 2,813)	0.8	0.8
MAL	2,557 (2,302, 2,837)	2,570 (2,239, 2,812)	2,635 (2,184, 2,857)	0.8	0.8
MIL	2,575 (2,199, 2,778)	2,522 (2,205, 2,768)	2,708 (2,383, 2,896)	0.2	0.3
MA	2,670 (2,417, 2,881)	2,562 (2,176, 2,797)	2,388 (1,675, 2,739)	<0.001	0.007
MI	2,718 (2,442, 2,891)	2,587 (2,276, 2,824)	2,442 (1,743, 2,820)	0.002	0.014

[1] Median (IQR)
[2] Kruskal-Wallis rank sum test
[3] False discovery rate correction for multiple testing

Abbreviations: AVC: Aortic valve closure; MAS: Mid-anteroseptal; MIS: Mid-infero-septal; MAL: Mid-anterolateral; MIL: Mid-inferolateral; MA: Mid-anterior; MI: Mid-inferior

E-Table 2: Comparison of characteristics between patients included and excluded from the longitudinal analysis

Characteristic	Excluded (n=115)[1]	Included (n=67)[1]	p-value[2]
Age (years)	65 (60, 72)	69 (61, 72)	0.2
Men	69 (62%)	47 (73%)	0.13
Body Mass Index (kg/m2)	29.5 (26.6, 34.7)	29.6 (26.0, 34.0)	0.7
Simplified Acute Physiology Score II	35 (29, 40)	36 (31, 42)	0.4
Sequential Organ Failure Assessment	4 (3, 5)	4.(3, 5)	0.6
Cardiopathy	9 (8.1%)	8 (13%)	0.3
Hypertension	64 (58%)	35 (55%)	0.7
COPD	1 (50%)	6 (75%)	>0.9
Chronic renal failure	10 (9.0%)	4 (6.3%)	0.5
Mean Blood Pressure (mmHg)	83 (75, 97)	98 (89, 107)	<0.001
Central Venous Pressure (mmHg)	8 (7, 10)	10 (8, 11)	0.3
PaO2/FiO2 (mmHg)	130 (97, 171)	86 (71, 124)	<0.001
Bicarbonates (mmol/L)	25.5 (23.5, 27.5)	24.4 (22.6, 25.8)	0.008
Creatinin (µmol/L)	66 (54, 86)	66 (59, 87)	0.5
Lactates (mmol/L)	1.50 (1.19, 1.90)	1.07 (0.92, 1.54)	<0.001
Platelets (MenG/L)	237 (182, 294)	265 (183, 322)	0.2
Vasopressors	10 (9.0%)	5 (7.8%)	0.8
Number of TEE	1(1, 1)	3 (2, 4)	<0.001
First day of TEE assessment	1 (1, 1)	1(1, 1)	0.5
Left Ventricular ejection fraction (%)	60 (52, 67)	57 (48, 65)	0.2
RV/LV end diastolic area ratio	0.80 (0.70, 0.90)	0.71 (0.70, 0.93)	0.9
End-Systolic Eccentricity Index	1.10 (1.00, 1.20)	1.10 (1.00, 1.20)	>0.9
TAPSE (mm)	22.0 (19.0, 24.4)	23.0 (19.0, 26.0)	0.3
Systolic right atrio-ventricular pressure gradient (mmHg)	29 (21, 36)	36 (29, 45)	<0.001
RV freewall strain (%)	26 (22, 31)	28 (25, 31)	0.11
ACP grade			0.13

Characteristic	Excluded (n=115)[1]	Included (n=67)[1]	p-value[2]
No ACP	81 (70%)	37 (56%)	
Moderate	21 (18%)	16 (24%)	
Severe	13 (11%)	13 (20%)	
**ICU mortality**	24 (21%)	32 (48%)	<0.001
[1] Median (IQR); n (%)			
[2] Wilcoxon rank sum test; Pearson's Chi-squared test; Fisher's exact test			

E-Table 3: Multivariate Time dependent Cox Model Regression using assessment only with conventional two-dimensional assessment alone and with the association of LV radial strain

	2D alone			2D and LV radial strain		
	**HR**[1]	**95% CI**[1]	**p-value**	**HR**[1]	**95% CI**[1]	**p-value**
**Acute cor pulmonale**			0.22			0.002
No Acute cor pulmonale	—	—		—	—	
Moderate grade 1	1.47	0.56, 3.83		2.1	0.73, 6.04	
Severe grade 1	1.57	0.43, 5.67		1.2	0.24, 5.96	
Severe grade 2	2.8	1.11, 7.09		6.27	2.28, 17.2	
**SAPS II (per point)**	0.99	0.94, 1.05	0.8	0.99	0.93, 1.05	0.69
**Age (per year)**	1.05	0.99, 1.11	0.07	1.04	0.98, 1.11	0.14

E-Figures

E-Figure 1: Flowchart of the study

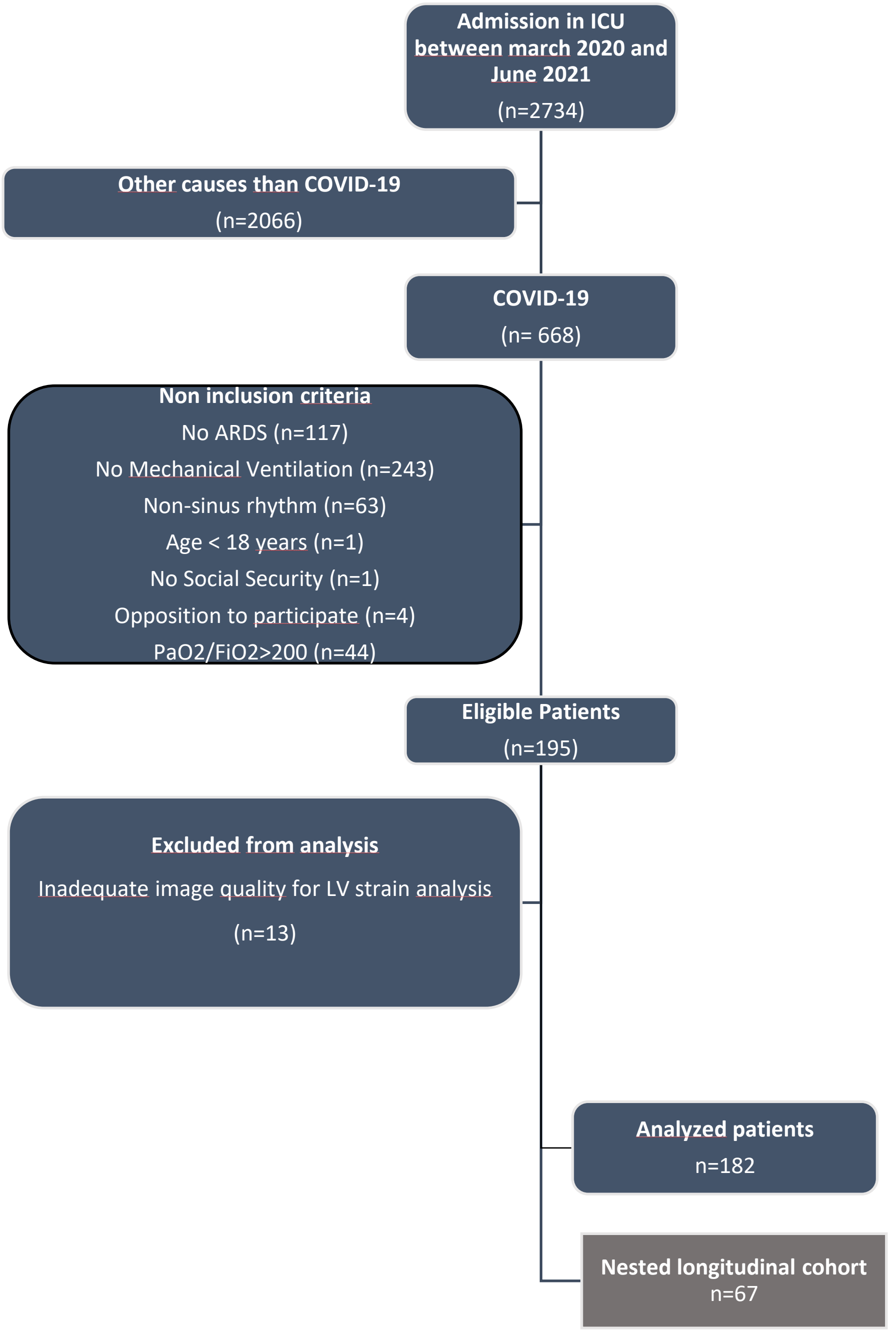

E-Figure 2: Boxplots with density plots comparing right ventricles parameters stratified by the grade of septal motion. P-value is provided only when significant and adjusted with Benjamini-Hochberg method to take account of the multiplicity of test.

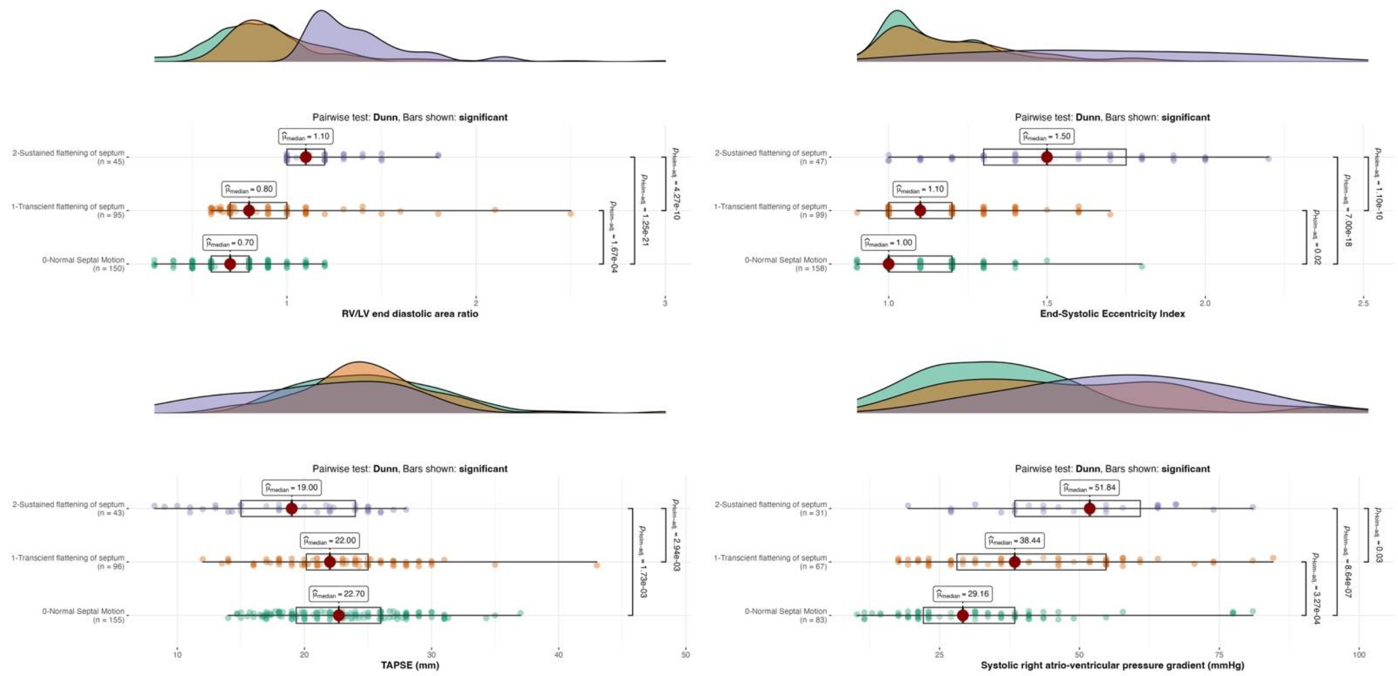

Abbreviations: RV: Right ventricle; LV: Left ventricle; TAPSE: Tricuspid annular plane systolic excursion

E-Figure 3: Boxplots with density plots comparing the time difference between time-to-peak and the time of aortic valve closure in each of left ventricular segment, and according to the grade of septal motion. In the left upper corner is a schematic representation of left ventricular segmentation used for the comparison. P-value is provided only when significant and adjusted with Benjamini-Hochberg method to take account of the multiplicity of test.

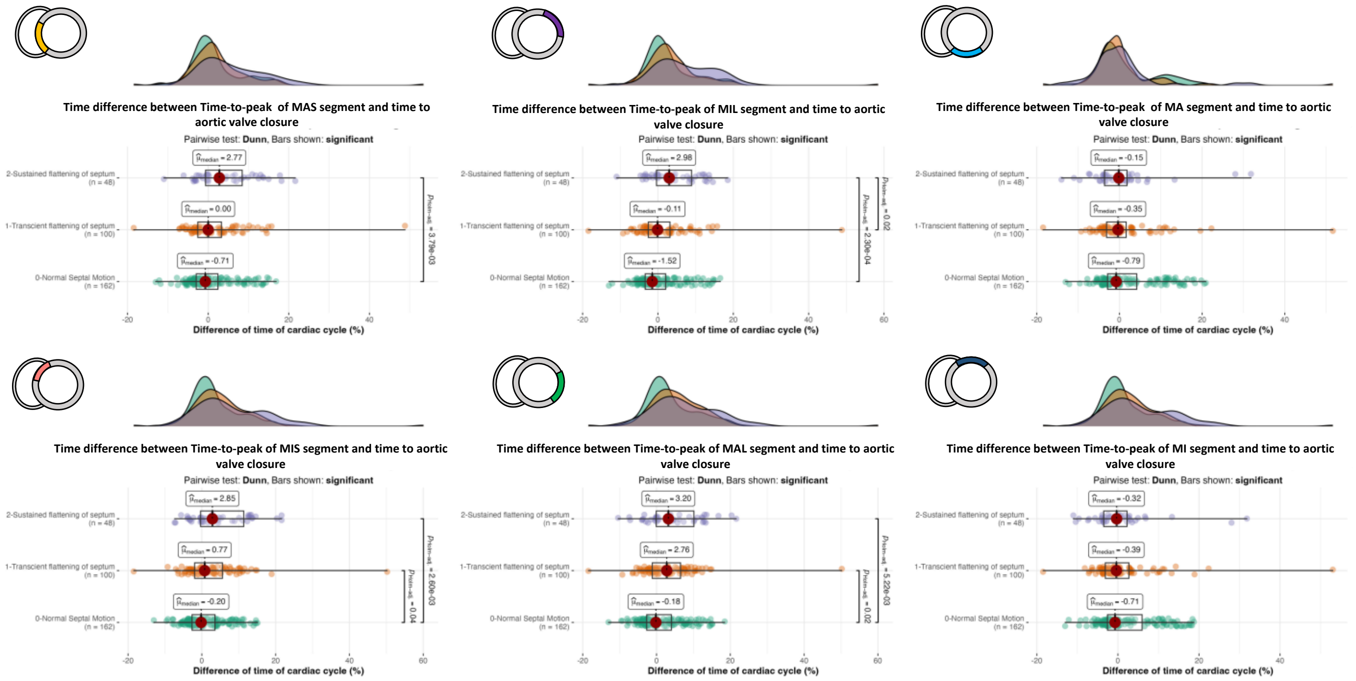

Abbreviations: AVC: aortic valve closure; MAS: Mid-anteroseptal; MIL: Mid-inferolateral; MA: Mid-anterior; MIS: Mid-infero-septal; MAL: Mid-anterolateral; MI: Mid-inferior

E-Figure 4: Boxplots with density plots comparing the time difference between time-to-peak of left ventricular septal segments and anterior or inferior segments in each grade of septal motion. In the left upper corner is a schematic representation of left ventricular segmentation used for the comparison. P-value is provided only when significant and adjusted with Benjamini-Hochberg method to take account of the multiplicity of test.

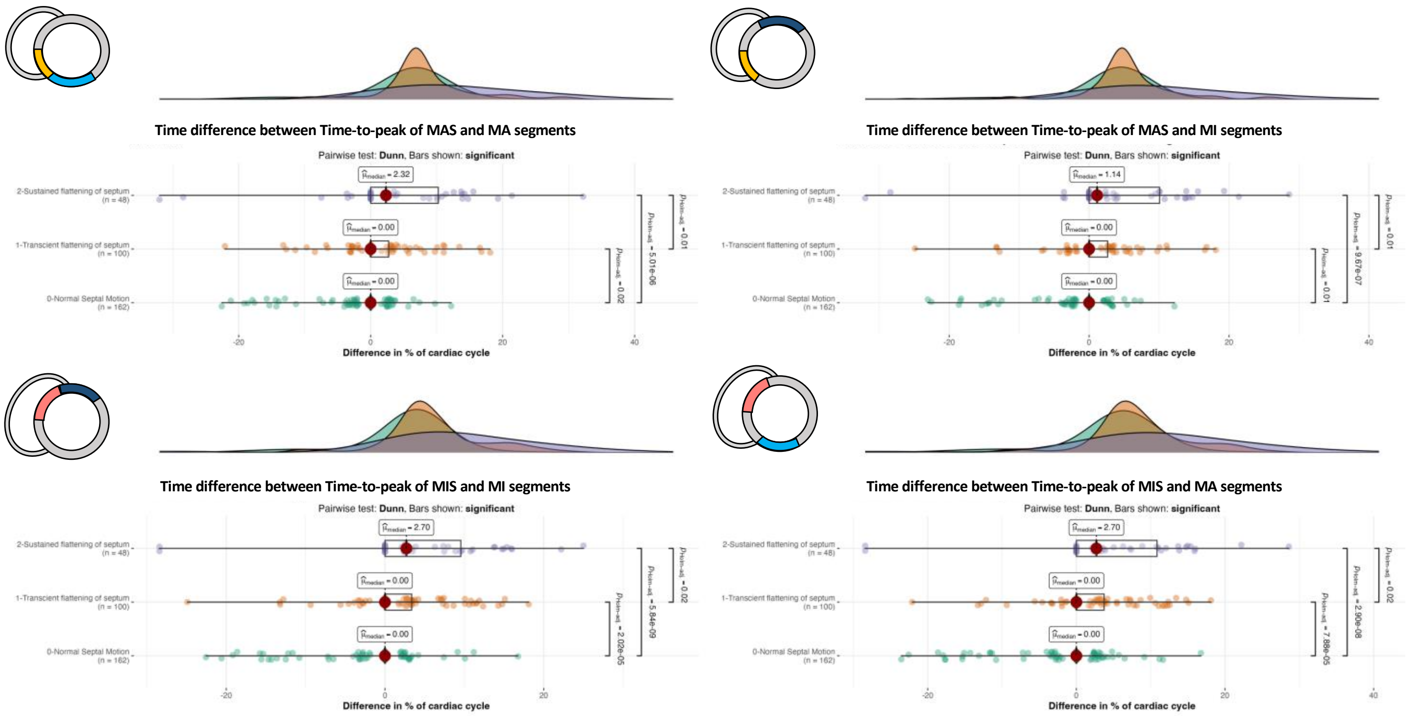

Abbreviations: MAS: Mid-anteroseptal; MA: Mid-anterior; MIS: Mid-infero-septal; MI: Mid-inferior

STROBE Statement

	Item No	Recommendation	Page No
**Title and abstract**	1	(*a*) Indicate the study's design with a commonly used term in the title or the abstract	1 and 3
		(*b*) Provide in the abstract an informative and balanced summary of what was done and what was found	3-4
**Introduction**			
Background/rationale	2	Explain the scientific background and rationale for the investigation being reported	6
Objectives	3	State specific objectives, including any prespecified hypotheses	6
**Methods**			
Study design	4	Present key elements of study design early in the paper	7
Setting	5	Describe the setting, locations, and relevant dates, including periods of recruitment, exposure, follow-up, and data collection	7
Participants	6	(*a*) *Cohort study*—Give the eligibility criteria, and the sources and methods of selection of participants. Describe methods of follow-up<br>*Case-control study*—Give the eligibility criteria, and the sources and methods of case ascertainment and control selection. Give the rationale for the choice of cases and controls<br>*Cross-sectional study*—Give the eligibility criteria, and the sources and methods of selection of participants	7
		(*b*) *Cohort study*—For matched studies, give matching criteria and number of exposed and unexposed<br>*Case-control study*—For matched studies, give matching criteria and the number of controls per case	
Variables	7	Clearly define all outcomes, exposures, predictors, potential confounders, and effect modifiers. Give diagnostic criteria, if applicable	8-11
Data sources/ measurement	8*	For each variable of interest, give sources of data and details of methods of assessment (measurement). Describe comparability of assessment methods if there is more than one group	8-11
Bias	9	Describe any efforts to address potential sources of bias	8-11
Study size	10	Explain how the study size was arrived at	10
Quantitative variables	11	Explain how quantitative variables were handled in the analyses. If applicable, describe which groupings were chosen and why	10
Statistical methods	12	(*a*) Describe all statistical methods, including those used to control for confounding	10-11
		(*b*) Describe any methods used to examine subgroups and interactions	10-11
		(*c*) Explain how missing data were addressed	10
		(*d*) *Cohort study*—If applicable, explain how loss to follow-up was addressed	NA

		Case-control study—If applicable, explain how matching of cases and controls was addressed<br>*Cross-sectional study*—If applicable, describe analytical methods taking account of sampling strategy	
		(*e*) Describe any sensitivity analyses	NA
**Results**			
Participants	13*	(a) Report numbers of individuals at each stage of study—eg numbers potentially eligible, examined for eligibility, confirmed eligible, included in the study, completing follow-up, and analysed	12
		(b) Give reasons for non-participation at each stage	12
		(c) Consider use of a flow diagram	E-Figure 1
Descriptive data	14*	(a) Give characteristics of study participants (eg demographic, clinical, social) and information on exposures and potential confounders	12 and Table 1
		(b) Indicate number of participants with missing data for each variable of interest	12-14
		(c) *Cohort study*—Summarise follow-up time (eg, average and total amount)	NA
Outcome data	15*	*Cohort study*—Report numbers of outcome events or summary measures over time	12-14
		Case-control study—Report numbers in each exposure category, or summary measures of exposure	
		Cross-sectional study—Report numbers of outcome events or summary measures	
Main results	16	(*a*) Give unadjusted estimates and, if applicable, confounder-adjusted estimates and their precision (eg, 95% confidence interval). Make clear which confounders were adjusted for and why they were included	12-14
		(*b*) Report category boundaries when continuous variables were categorized	11-12
		(*c*) If relevant, consider translating estimates of relative risk into absolute risk for a meaningful time period	NA
Other analyses	17	Report other analyses done—eg analyses of subgroups and interactions, and sensitivity analyses	14
**Discussion**			
Key results	18	Summarise key results with reference to study objectives	15
Limitations	19	Discuss limitations of the study, taking into account sources of potential bias or imprecision. Discuss both direction and magnitude of any potential bias	17-18
Interpretation	20	Give a cautious overall interpretation of results considering objectives, limitations, multiplicity of analyses, results from similar studies, and other relevant evidence	15-18
Generalisability	21	Discuss the generalisability (external validity) of the study results	17-18
**Other information**			
Funding	22	Give the source of funding and the role of the funders for the present study and, if applicable, for the original study on which the present article is based	2

*Give information separately for cases and controls in case-control studies and, if applicable, for exposed and unexposed groups in cohort and cross-sectional studies.

Note: An Explanation and Elaboration article discusses each checklist item and gives methodological background and published examples of transparent reporting. The STROBE checklist is best used in conjunction with this article (freely available on the Web sites of PLoS Medicine at http://www.plosmedicine.org/, Annals of Internal Medicine at http://www.annals.org/, and Epidemiology at http://www.epidem.com/). Information on the STROBE Initiative is available at www.strobe-statement.org.

Supplementary references

1. Vignon P. Assessment of Pulmonary Arterial Pressure Using Critical Care Echocardiography: Dealing With the Yin and the Yang?*. Crit Care Med. 2019;47:126–8.

2. Lang RM, Badano LP, Mor-Avi V, Afilalo J, Armstrong A, Ernande L, et al. Recommendations for Cardiac Chamber Quantification by Echocardiography in Adults: An Update from the American Society of Echocardiography and the European Association of Cardiovascular Imaging. J Am Soc Echocardiogr. 2015;28:1-39.e14.

3. Antoine Humeau / qlabRaw2RectangularData · GitLab [Internet]. GitLab. 2023 [cited 2023 Aug 23]. Available from: https://gitlab.com/antoinehumeau/qlabraw2rectangulardata

4. Sjoberg D D, Whiting K, Curry M, Lavery J A, Larmarange J. Reproducible Summary Tables with the gtsummary Package. R J. 2021;13:570.

5. Gu Z. Complex heatmap visualization. iMeta. 2022;1:e43.

6. Brunson JC. ggalluvial: Layered Grammar for Alluvial Plots. J Open Source Softw. 2020;5:2017.

7. Patil I. Visualizations with statistical details: The “ggstatsplot” approach. J Open Source Softw. 2021;6:3167.
